# Health Needs and Challenges in Preventing Secondhand Smoke Exposure Among Rural Adolescents: Implications for Innovative Health Education

**DOI:** 10.1097/jnr.0000000000000724

**Published:** 2026-01-26

**Authors:** Yu-Shuan WAN, Tung-Jung HUANG, Chia-Hao CHANG, Mei-Yen CHEN

**Affiliations:** 1 Department of Nursing, Chang Gung University of Science and Technology, Chiayi, Taiwan; 2 Department of Internal Medicine, Chang Gung Memorial Hospital, Yunlin, Taiwan; 3 Department of Respiratory Care, Chang Gung University of Science and Technology, Chiayi, Taiwan; 4 Department of Cardiology, Chang Gung Memorial Hospital, Chiayi, Taiwan; 5 Graduate Institute of Gerontology and Health Care Management, Chang Gung University of Science and Technology, Taoyuan, Taiwan; †Contributed equally

**Keywords:** adolescent, secondhand smoke, innovative strategies, parental smoking, moral dilemmas

## Abstract

**Background:**

Adolescents exposed to secondhand smoke (SHS) and those living with parents who smoke are at higher risk for tobacco use and adverse health outcomes. School nurses are expected to support smoke-free policies on campuses and play a prominent role in health promotion. However, few nursing studies on SHS exposure prevention have included recommended strategies for advising family smokers (AFS).

**Purpose:**

This study was developed to examine the prevalence of SHS exposure among adolescents in rural areas and assess the impact of implementing a pilot program that incorporates innovative strategies for AFS.

**Methods:**

A school-based, educational intervention study was conducted in four middle schools in western coastal Yunlin County between March and September 2023. Innovative strategies for AFS were integrated into the school’s health education curricula using a 6-hour secondhand smoke prevention program. Data were collected using self-administered, anonymous questionnaires. Descriptive and inferential statistical analyses were employed to examine the factors and barriers associated with AFS.

**Results:**

Of the 1,202 adolescents who participated at baseline, 806 (51.4% boys; mean age=13.5 years) reported living with a smoker at home (67%). Among these 806, 91.4% reported family members smoked indoors, and, after completing the AFS program, 46.9% reported having attempted to advise their family members to quit smoking or smoke outdoors. The factors identified as significantly associated with AFS included being female, practicing avoidance behaviors, and witnessing smoking on campus. Only 8.1% felt their advice was successful, with common challenges including parental refusal, being scolded, and feelings of hopelessness. Also, many of the participants reported experiencing moral dilemmas, particularly with regard to cultural values such as filial piety, when advising parents.

**Conclusions/Implications for Practice:**

Exposure to secondhand smoke among adolescents living in rural areas is a serious issue. Teaching them strategies on how to advise family smokers may help reduce home exposure, particularly among boys. However, in Eastern cultures, advising parents about smoking presents moral challenges due to cultural values. Regulatory changes are needed to protect youths from secondhand smoke at home.

## Introduction

Research has shown that adolescents exposed to secondhand smoke (SHS) and those living with parents who smoke are at higher risk of tobacco use and adverse health outcomes (World Health Organization [WHO], 2024a). Primary care nurses, including school nurses, are expected to support smoke-free policies on campuses (Health Promotion Administration [HPA], 2024; Sarna et al., 2019). The International Council of Nurses (ICN) and nursing educators regularly advocate for the integration of tobacco prevention, cessation, and SHS avoidance into nursing practice (ICN, 2024; Pereira et al., 2023). Aligning with the United Nations’ Sustainable Development Goals (SDGs), and with goals 3 (good health and well-being) and 10 (reduced inequalities) in particular, is central to the nursing profession (Dong et al., 2022; Fields et al., 2021; ICN, 2024). Thus, it is essential that primary care nurses initiate health-promoting programs for adolescents, especially in disadvantaged areas.

Despite increasing public awareness about the dangers of SHS, this issue remains largely overlooked in current health and environmental policies (Arfaeinia et al., 2023). Taiwan’s Tobacco Hazards Prevention Act prohibits smoking in indoor public areas for those under 18 and mandates that local hospitals and health centers offer free smoking cessation programs (HPA, 2024; Tobacco Hazards Prevention Act, 2023). However, this law does not regulate parental smoking at home with children, allowing many parents and relatives to continue smoking around youths. This challenge persists globally, as many countries struggle to mitigate adolescents’ SHS exposure at home (Azagba et al., 2020; Hanafin et al., 2021; WHO, 2024a, 2024b). A critical analysis of studies of school-based or family-involved smoking prevention programs implemented in various countries (Ma et al., 2021; Pawils et al., 2023; Ylitörmänen et al., 2023) found that few incorporated direct strategies using structured frameworks aimed at training adolescents to advise family smokers. Most of the school-based tobacco prevention programs in these studies focused primarily on raising awareness of the hazards of smoking and developing refusal skills, and rarely provided adolescents with practical tools to engage family members in smoking cessation or SHS avoidance. Furthermore, few nursing studies have explored nurse-led SHS prevention programs that integrate strategies for advising family smokers (AFS) and promoting protective measures.

Adolescents living with family smokers are at risk of exposure to SHS, which comprises a mix of sidestream and mainstream smoke as well as thirdhand smoke (THS). THS refers to pollutants lingering on surfaces in indoor environments (Arfaeinia et al., 2023; Mariano et al., 2022). SHS and THS contain harmful chemicals that can pollute the air and settle on surfaces, posing serious health risks. Empirical evidence shows nicotine to be highly addictive and particularly detrimental to the biopsychosocial health of adolescents (Alnajem et al., 2020; WHO, 2024a). Adolescents exposed to passive smoking from friends or family members are significantly more likely to engage in tobacco use and unhealthy behaviors, to experience poor psychological well-being, and to face academic challenges (Choi et al., 2020; Lastunen et al., 2017; Liang et al., 2022). Research findings indicate smoking often begins around age 13 and is a habit strongly influenced by peers and caregivers (Hilli & Pedersen, 2021; Laverty et al., 2019; Liang et al., 2022). High rates of adult smoking at home have been observed in Taiwan’s western coastal areas (>60%) and the United States, with people in rural areas having limited access to cessation or health programs for rural residents (Chen, 2021; Dai et al., 2021; HPA, 2024). In addition, adolescent smoking rates exhibit significant geographic disparities, with a higher prevalence in rural than urban settings (Dai et al., 2021; Kim & Selya, 2022). Therefore, nurses working at middle schools play a vital role in protecting adolescent health by implementing early detection measures, smoking prevention programs, and strategies to reduce exposure to secondhand smoke (Hilli & Pedersen, 2021; Pawils et al., 2023).

Due to limited access and cultural sensitivities surrounding parental authority and smoking behaviors—particularly within rural Taiwanese families--direct intervention with family smokers has generally not been feasible as a part of school-based activities. Thus, this study was designed to strategically empower adolescents, who are often passive recipients of SHS, to initiate conversations and influence change within their households. To the knowledge of the authors, few nursing-led interventions have utilized scenario-based roleplay models to empower adolescents in Eastern cultures, where familial hierarchy and filial piety significantly shape communication dynamics. This highlights the innovative nature of the adolescent-centered approach taken in this study within a rural Taiwanese context. Although current health policies do not adequately address SHS exposure at home (Tobacco Hazards Prevention Act, 2023), school settings offer an opportunity for nurses to advocate for adolescent health by teaching innovative strategies to encourage family smokers to quit or refrain from smoking inside the home.

## Methods

### Study Design and Participants

A school-based, educational intervention study was conducted at four middle schools in Yunlin County in southwest Taiwan between March and September 2023. This study was carried out in collaboration with four school nurses as well as with two public health nurses working in the targeted area. The inclusion criteria were adolescents enrolled in the 7th to 9th grades at one of the four participating middle schools in Yunlin County who had returned consent forms signed by both the student and a guardian. The exclusion criteria included participants who provided incomplete data or were absent for more than two sessions of the program. Based on G*Power 3.1, the priori power analysis for logistic regression conducted with an assumed medium effect size (*OR*=1.5), α=.05, and power=0.90 (Faul et al., 2009) indicated a minimum of 670 participants was required. An initial sample of 1,202 participants was recruited, of which 67.1% (*n*=806) reported living with at least one family member who smoked. These 806 participants proceeded to the second phase of the study and completed the educational intervention sessions. The size of this sample provided sufficient power to detect statistically significant associations.

### Procedure and Ethical Considerations

The research protocol was reviewed and approved by the institutional review board of Chang Gung Memorial Hospital (IRB No. 202200201A3). Before participation, both the participants and one of their parents/legal guardians received detailed information sheets (provided inside an envelope) outlining the study purpose, procedures, confidentiality protections, and right to withdraw at any time without penalty. Written informed consent was obtained from at least one parent or guardian, and assent was simultaneously obtained from each participant. All of the collected data were deidentified and securely stored to ensure participant confidentiality.

With regard to the AFS-enhanced SHS program, each class (~20–25 students) received four consecutive weekly sessions of 1.5 hours each for a total of 6 hours, which were credited toward their school’s health education curriculum. This program was designed with the following objectives: (1) improve understanding of secondhand and thirdhand smoke (SHS/THS) and related health effects, (2) develop skills useful in advising family smokers using the five-step (plan, discuss, contact, assistance, and protective strategy, PDCAP), and (3) increase confidence in initiating respectful, culturally appropriate discussions with family members who smoke.

To standardize the instruction in this program and ensure content consistency, all four nurse instructors followed a standardized protocol, including using pre-approved PowerPoint slides and teaching videos developed and reviewed by the research team based on the Health Promotion Administration guidelines (HPA, 2024). Before the intervention, all of the instructors participated in a 3-hour training workshop led by the principal investigator to ensure uniform delivery and understanding of the PDCAP framework.

Each session was structured as follows: Session 1: Introduction to tobacco-related harms and SHS and THS concepts; Session 2: PDCAP strategy introduction and communication techniques; and Sessions 3 and 4: Scenario-based roleplay, rehearsal, peer feedback, and reflection. Scenario-based roleplay was incorporated as a teaching strategy for AFS, with students instructed to imagine discovering a parent smoking in the house (e.g., “One day, you wake up and find your dad smoking in the living room”). The scenario included two phases: (1) Role assignment, with students acting as family members (e.g., student A as the smoking father, student B as the mother, student C as a sibling, and student D as the adolescent addressing the situation) and (2) Rehearsal and feedback to refine responses.

The research team included four nurse educators, each of whom was certified at the highest smoking cessation instructor certification level (Level 3). To ensure content validity, the content validity index (CVI) was applied to the educational materials and semi-structured questionnaire. Five experts (two physicians specializing in chest and family medicine, two PhD nursing faculty, and one health educator) assessed the relevance of each item on a scale ranging from 1 (*not relevant*) to 3 (*very relevant*), yielding a CVI of .89 (range: .81–.99).

Unlike traditional school-based tobacco prevention programs, the AFS strategy teaches adolescents to communicate with empathy and respect using the five-step PDCAP model, which was developed by the current research team based on standard nursing procedures, culturally relevant communication principles, and family dynamics common in East Asian cultural settings. The AFS framework encourages students to collaborate with supportive family members (e.g., siblings, grandparents) and provides resources such as videos and cessation service information.

1. Plan: Choose a peaceful family time, such as the weekend or a holiday, and prepare SHS teaching materials.
2. Discuss: Share your plan with a trusted family member, for example, your mother, siblings, or grandmother, for support.
3. Contact: Approach the smoker (e.g., father or grandfather) in a friendly, polite manner. Begin with a smile and express appreciation for something positive he has done. Then, (a) discuss the health risks of tobacco, SHS, and THS exposure (e.g., asthma, lung illness, hypertension, heart disease) and (b) politely request that he smoke outside, consider quitting, or reduce consumption.
4. Assistance: Offer support by (a) providing useful smoking cessation resources such as educational materials from a government website (HPA, 2024) and (b) encouraging them to join a smoking cessation program at a local health center or clinic.
5. Protective strategies: If smoking occurs around you, express your discomfort, move away, or open a door or window for ventilation.

### Measurement

Baseline data were collected 1 week before the first session, and follow-up data were collected 5 months after program completion. The self-developed questionnaire included the following eight items: (a) Do any family members you live with smoke? (Yes/No); (b) Do your family members smoke around you? (Never/Sometimes/Usually); (c) How harmful do you think secondhand smoke is to health? (I don’t think so/Somewhat/Highly); (d) Have you tried avoidance behaviors to prevent inhaling secondhand smoke? (Never/Sometimes/Usually); (e) Have you used cigarettes or e-cigarettes in the past month? (Yes/No); (f) Have you observed anyone smoking on the school campus? If so, who? (g) Have you tried to advise your family members to quit or avoid smoking? (Yes/No); If yes, briefly describe the main strategies you used; if not, briefly explain the reasons; and (h) Describe any experiences or barriers you encountered while using these strategies. The questionnaire was printed on a single A4 page and required 15–20 minutes to complete. At the first stage, participants were asked to complete items 1–6 on a single A4 page, along with basic sociodemographic information. At the second stage, participants were asked to respond to items 7 and 8, which were presented in a 3-column, 6-row format on an A4 sheet. This sheet also included a description of the PDCAP steps and a blank space.

The five-month follow-up period (March to September) was chosen based on academic scheduling and practical considerations. This duration allowed sufficient time for students to internalize and apply the learned AFS strategies in real-life family settings. Importantly, this period included summer vacation, during which adolescents generally spend more time at home and have greater opportunities to engage with family members, making it favorable to observe behavioral changes.

### Data Analysis

Microsoft Excel (2019) was used to record participants’ responses regarding their experiences and barriers in advising family smokers. IBM SPSS Statistics version 26 (IBM Corp., Armonk, NY, USA) was used for descriptive and inferential statistics. Categorical and continuous demographic variables and advising behaviors were analyzed using frequencies and percentages. The written descriptions provided by the participants were organized and categorized by two researchers, with the first author conducting the initial organization and categorization, followed by revisions made in response to the expert review. Specifically, the written strategies used by the participants and the barriers encountered when advising family smokers were categorized into common themes. Logistic regression analysis was performed to examine the significant factors associated with adolescents advising family smokers. All of the variables found to be significantly associated with the outcome in the univariate analysis were included in the multivariable model. Potential interaction effects (e.g., gender × avoidance behaviors, SHS perception × school observation) were tested but found not statistically significant and thus excluded from the final model. Model fit was assessed using the Hosmer and Lemeshow goodness-of-fit test, and *OR* and 95% confidence intervals (CIs) were reported as effect size measures for each independent variable. A *p* value of<.05 was considered statistically significant for all analysis results.

## Results

Of the 1,282 students at the four targeted middle schools (7th to 9th grade), 1,202 enrolled as participants in this study (response rate: 93.7%). Of these, 67.1% (*n*=806) reported living with at least one family member who smoked and subsequently participated in the second part of the study and completed the AFS educational intervention sessions. As shown in Table 1, slightly over half of the participants were male (*n*=414, 51.4%). The mean age was 13.5 years (range: 12–15, *SD*=1.2). Most (85%) recognized the health risks of SHS, 9.8% reported being a current or former smoker, and 6.3% reported e-cigarette use (predominantly male participants). In addition, 51.6% observed smokers on their school campus, with 25.2% identifying these smokers as teachers. After completing the nurse-led AFS program, 46.9% (*n*=378) reported providing smoking-danger/cessation advice to family members who smoked.

**Table 1:** Demographic Characteristics of Participants Living With Family Smokers (*N*=806)

Variable	*n* (%)
Gender	
Boy	414 (51.4)
Girl	392 (48.6)
Age (years; mean ± *SD*, range)	13.5±1.2 (12–15)
Grade	
7th	282 (35.0)
8th	247 (30.6)
9th	277 (34.4)
Family members smoke in the home	
Never	69 (8.6)
Sometimes	416 (51.6)
Usually	321 (39.8)
Health harms of secondhand smoke	
I don’t think so	8 (1.0)
Possibly	110 (13.6)
Yes	688 (85.4)
Avoidance behavior	
Never	72 (8.9)
Sometimes	326 (40.4)
Usually	408 (50.6)
Advising family smokers	
Yes	378 (46.9)
No	428 (53.1)
Consumer of paper cigarettes	
Never	727 (90.2)
Yes (current and former) ^a^	79 (9.8)
Consumer of electronic cigarettes	
Never	755 (93.7)
Yes ^b^	51 (6.3)
Observed smokers at school	
Never	390 (48.4)
Yes ^c^	416 (51.6)
Visitors	284 (35.2)
School teachers	203 (25.2)
Classmates or students	98 (12.2)
School guards	37 (4.6)

^a^ Male gender (χ^2^=4.08, *p*<.05); ^b^ male gender (χ^2^ =13.36, *p*<.001); ^c^ multiple choice.

Being female (*p*<.001), being aware of the health risks of SHS (*p*<.05), practicing avoidance behaviors in response to SHS from family smokers (*p*<.001), and observing smokers on campus (*p*<.001) were identified as significantly associated with advising behaviors toward family smokers (Table 2). Based on the findings of the logistic regression analysis, the most important factors associated with advising behaviors were being female (adjusted odds ratio [a*OR*]=2.23, confidence interval [CI]=[1.66, 2.97]), practicing SHS avoidance behaviors (a*OR*=1.8, CI=[1.34, 2.40]), and observing smokers on campus (a*OR*=1.59, CI=[1.19, 2.13]; Table 3).

**Table 2:** Factors Associated With Advising Family Smokers (*N*=806)

Variable	Advising Family Smokers, *n* (%)		χ^2^	*p*
	Yes (*n*=378)	No (*n*=428)		
Gender			29.04	<.001
Girl	222 (58.7)	170 (39.7)		
Boy	156 (41.3)	258 (60.3)		
Grade			3.37	.185
1st	143 (37.8)	139 (32.5)		
2nd	116 (30.7)	131(30.6)		
3rd	119 (31.5)	158 (36.9)		
Health harms of secondhand smoke			6.07	<.050
May be not/not sure	43 (11.4)	75 (17.5)		
Firmly sure	335 (88.6)	353 (82.5)		
Consumer of paper cigarettes			2.06	.151
Never	347 (91.8)	380 (88.8)		
Yes (current and former)	31 (8.2)	48 (11.2)		
Consumer of electronic cigarettes			0.01	.981
Never	354 (93.7)	401 (93.7)		
Yes (current and former)	24 (6.3)	27 (6.3)		
Family members smoke around you			0.25	.618
Never and sometimes	224 (59.3)	261 (61.0)		
Yes (usually)	154 (40.7)	167 (39.0)		
Avoidance behaviors			19.97	<.001
Never and sometimes	155 (41.0)	243 (56.8)		
Yes (usually)	223 (59.0)	185 (43.2)		
Observed smokers at school			7.13	<.001
Never	164 (43.4)	226 (52.8)		
Yes	214 (56.6)	202 (47.2)		

**Table 3:** Logistic Regression of Factors Associated With Advising Family Smokers

Variable	*B*	*SE*	*p*	a*OR*	95% CI	
					Lower	Upper
Gender (ref=boy)	0.80	0.15	<.001	2.23	1.66	2.97
Health harm of SHS (ref=not sure)	0.39	0.22	.064	1.49	0.98	2.27
Avoidance behaviors (ref=low)	0.59	0.15	<.001	1.80	1.34	2.40
Observed smokers at school (ref=never)	0.47	0.15	<.01	1.59	1.19	2.13

*Note.* Hosmer and Lemeshow test: χ^2^=4.982, Significance: 0.662. Ref=reference group; SHS=secondhand smoke; CI=confidence interval; a*OR*=adjusted odds ratio.

The strategy most commonly used by adolescents in advising family smokers was “discussion,” employed by 74.3% (*n*=281; Table 4). Examples include negotiating with their father, reminding them of the health risks of smoking, politely advising or suggesting that smoking be done outdoors. The 15.9% (*n*=60) who reported using “assistance” strategies did so by referencing internet resources, showing family members online videos about quitting, encouraging visits to smoking cessation clinics, and bringing home posters from school or local health centers.

**Table 4:** Most Commonly Used Strategies in Advising Family Smokers (*N*=378)

Strategy/Example	*n* (%)
Discussion Remind of the health harms of cigarette smoking and secondhand smoke. Politely advise or suggest verbally. Say that secondhand smoke can harm others. Ask courteously that smoking be done outside. Negotiate with daddy. Ask Mom (sister) to help.	281 (74.3)
Assistance Show them online videos about quitting smoking. Use the media. Assist them sign up for smoking cessation courses. Assist them sign up for smoking cessation clinics. Assist them register at smoking cessation clinics. Set up penalty rules at home. Take a poster home from school or health center. Chew gum or do something of interest (to distract from smoking).	60 (15.9)
Protective strategies “It stinks,” and use both hands to express dislike. “Oh no, I don’t like it,” and run away. I beg them to quit. Run away when they come. Throw away the cigarette. Hide the cigarette box.	37 (9.8)

As shown in Table 5, 54.2% of the sample experienced refusal, 22% were scolded, and 15.6% encountered excuses from family smokers when providing advice on smoking. Examples of responses include: “I tried many times, but he always says it’s difficult” “He says smoking is for work” “He says he feels bad if he doesn’t smoke” and “Children shouldn’t meddle in adult affairs.” However, 8.1% felt their efforts were met with success, with comments such as: “Yeah, he changed his habit and now smokes outside” “My father listened and reduced the amount” “He quit smoking” and “Mom says he no longer smokes indoors—yeah!”

**Table 5:** Experiences and Barriers Encountered While Advising Family Smokers (*N*=378)

The Most Commonly Used Text Descriptions	*n* (%)
Refused They always said no. They didn’t listen to me. I tried many times, but he always says it is difficult. I was rejected and felt very sad. I felt hopeless. The addiction is too strong to quit. They always say yes yet continues to smoke. My grandfather agreed initially, but then he went back to smoking again.	205 (54.2)
Scolded He said: Children are forbidden to interfere in adult affairs… He says that children have ears but no mouth. He said, you don’t need to care about me… He always says that children shouldn’t meddle in adult affairs. I was scolded. He scolds me… He stared at me…	83 (22.0)
Excuse He always said he was under pressure… He always says that smoking is for work. Felt blue if no smoking, he said… He was tempted by his friends who smoked. He always says he feels bad if he doesn’t smoke. He quit for a while but tempted by his friend…	59 (15.6)
Yeah, success Yeah, he changed his smoking habit. My grandfather listens to me; he reduced the amount, he smokes outside. My father is so cute, he listened to my advice. He smokes outdoors now. Mom said he doesn’t smoke in the house. Mom says he is no longer smoking indoors, yeah! He has reduced consumption so far, yeah! Finally, he quit, I felt so happy.	31 (8.1)

## Discussion

This study underscores the urgent need to equip adolescents with additional resources and strategies to reduce SHS exposure at home and promote smoke-free school environments.

### High Prevalence of Parental Smoking at Home and Its Consequences

Nearly two-thirds of the participants reported living with a smoker, with over 90% of these smokers smoking inside the home. Although the SHS prevalence in this study is lower than the 75% reported in a study from China (Yang et al., 2021), it is higher than rates reported by other middle school students in Taiwan (30%–33%; HPA, 2024) as well as their counterparts in the United States (Choi et al., 2020) and the global average, which identifies ~33%–40% of children as exposed to SHS (Arfaeinia et al., 2023; Ma et al., 2021).

Notably, nearly one in 10 of the participants (most of whom were males) self-reported as a current or former (within the past month) smoker. Nationwide, adolescent smoking rates in middle school have decreased from 7.8% to 2.8% (HPA, 2024). Percentages from other countries include 11.3% in the United States (Gentzke et al., 2022), 13.8% in the United Kingdom (Laverty et al., 2019), and 4.1% in Canada (Liang et al., 2022), where males also dominate in this category (Azagba et al., 2020). Moreover, the rate found in this study is higher than that found across 42 other countries (7.7%–10%; WHO, 2024a; Ylitörmänen et al., 2023). Social determinants of health provide a possible reason for these differences, reflecting the conditions in which people are born, grow, work, live, and age, which significantly influence health disparities and life expectancy (WHO, 2024b).

The findings of this study align with previous research that shows significant disparities in adolescent smoking rates between rural and urban areas (Dai et al., 2021; Kim & Selya, 2022). Friends, parents, and caregivers have been widely identified as heavily influencing adolescent tobacco and e-cigarette use (Lastunen et al., 2017; Laverty et al., 2019; Yang et al., 2021). For example, in Ireland, the rise in e-cigarette ever-use from 23% in 2015 to 37% in 2019 and in current use from 10% to 18%, respectively, was largely explained by the influence of peers and family (Hanafin et al., 2021). Although this study reported a relatively low (6.3%) combined rate of ever and current e-cigarette use, the rising trend is concerning (Azagba et al., 2020; Liang et al., 2022), warranting close attention from middle school staff. Idioms such as “like father, like son” and “birds of a feather flock together” highlight the influence of parental and peer smoking on adolescents, especially boys. Primary care nurses are crucial in safeguarding adolescent health through the early detection and prevention of cigarette smoking and SHS exposure on school campuses. This may be achieved using a multidisciplinary approach that involves advocacy on family-teacher days, collaboration with school principals, and engagement with local policymakers.

### Concerns and Moral Dilemmas in Advising Family Smokers: Signs of Hope

While most of the participants recognized the “health harm of SHS exposure,” only half consistently practiced avoidance behaviors to prevent inhaling secondhand smoke. Nearly half of the participants attempted to advise smoking family members (mainly fathers) using the strategies they learned, indicating the program succeeded in empowering some of the participants to address SHS exposure at home. However, more than half that did not, indicating the influence of practical barriers such as fear of confronting family members, cultural expectations (e.g., filial piety), and uncertainties regarding how to communicate effectively. Some of the participants expressed hesitation, stating, for example, “I don’t know how to convey it (SHS avoidance messages)” and “I’m afraid of starting the conversation” even after learning SHS prevention strategies.

In Eastern societies, adolescents, especially boys, may find it challenging to advise their fathers to quit smoking or avoid smoking in the home due to cultural influences such as filial piety and patriarchal values. Over half of the participants reported encountering refusal, with family smokers often ignoring their advice, making excuses, or expressing difficulty in quitting—leaving the adolescents feeling sad, hopeless, and rejected. In addition, 22% of the participants were scolded for “interfering in adult affairs,” highlighting a cultural barrier in which filial piety discourages children from questioning or advising their elders. Hence, developing family-inclusive SHS prevention programs, school-led family health campaigns, and policy incentives for parents who participate in cessation programs is recommended. Also, future interventions may include (1) roleplay exercises tailored for high-resistance scenarios; (2) communication coaching for adolescents and family members; and (3) involvement of school teachers and local health units to reinforce messages across settings.

Despite these challenges, 8.1% (*n*=31) of the participants reported success in terms of some family members reducing cigarette use, smoking outdoors, or quitting altogether. These findings highlight the need for further exploration of the specific challenges adolescents encounter when advising family smokers, and suggest that additional support or tailored strategies may help more youths feel comfortable and confident in initiating these challenging conversations. Furthermore, based on the qualitative responses, the most commonly used and effective strategies among the 8.1% who reported achieving success included polite negotiation, sharing SHS-related health information learned from school, using supportive family members (e.g., mothers or siblings) to mediate, and recommending local smoking cessation resources. These align with the “Discussion” and “Assistance” phases of the PDCAP model, indicating multistep, respectful communication paired with practical help may represent a relatively more persuasive approach.

Although it is encouraging that nearly half of the participants (46.9%) attempted to advise family members following the AFS program, the low rate of success achieved (8.1%) indicates significant barriers remain in translating knowledge into behavioral change within family dynamics. This disparity highlights the complexities of adolescent–parent interactions, especially in the context of deeply rooted cultural values such as filial piety and respect for elders, which may inhibit assertive communication. Furthermore, the reluctance of adults to accept advice from children, compounded by power imbalances and emotional resistance to quitting, underscores the need for family-based interventions that directly engage parents or caregivers. Future programs should consider integrating parallel educational components for family members along with stronger reinforcement mechanisms such as motivational interviewing, digital feedback tools, and ongoing counseling to improve the receptivity and responsiveness of smoking adults to adolescent-initiated conversations. Addressing this gap will be crucial to enhancing the real-world effectiveness of youth-led secondhand smoke prevention strategies.

In Park and Chang (2020), which used digital media to help better empower adolescents in smoking prevention programs, both quantitative and qualitative evidence were found to support the effectiveness of co-producing anti-smoking videos as a health education method, particularly among those living in low-income areas. In future studies, the potential of using photovoice and visual technologies (e.g., smartphones; Dong et al., 2022; Laholt et al., 2019) combined with scenario-based roleplay to further enhance the skills of adolescents in persuading family members who smoke may be explored. For example, in a study conducted in Norway, public health nurses used visual methods in health dialogue with adolescents, finding photovoice to be useful in health promotion activities targeting adolescents (Laholt et al., 2019). Moreover, in Ireland, Goodwin et al. (2023) suggested that, in the realm of nursing care, traditional print media is relatively ineffective and film/TV is relatively more effective in influencing patient perceptions of mental health services. Thus, a multimedia approach to smoking prevention education may be more effective in influencing adolescent populations. These approaches may empower adolescents with creative and culturally relevant tools for initiating difficult conversations with family smokers and may be integrated into future iterations of school-based SHS prevention programs.

### Establishing a Smoke-Free Campus Environment is Urgent

Friends, parents, and public smoking have been previously identified as key risk factors for adolescent smoking behaviors, particularly among those living in rural areas (Roberts et al., 2020; Singh et al., 2021). Environmental factors, including home SHS exposure, peer smoking, SHS exposure outside school, and SHS on school grounds, significantly influence adolescent smoking habits (Liang et al., 2022). The finding in this study that 25.2% of the smokers observed by the participants at school were teachers is particularly concerning. Teachers serve as role models, and their smoking behavior may convey conflicting messages to students about the acceptability of tobacco use. The findings of previous studies indicate that the exposure of adolescents to adult smoking, especially from influential figures like teachers, increases their likelihood of smoking initiation and reduces their adherence to smoke-free policies (Roberts et al., 2020; Singh et al., 2021). Therefore, stricter enforcement of smoke-free campus regulations (HPA, 2024) and targeted teacher education may represent crucial components in creating a consistent and supportive environment for tobacco prevention among adolescents.

Nursing scholars have recently emphasized the importance of schools as environments for fostering healthy habits in students, with public health nursing rooted in public health principles and core nursing values (Clancy et al., 2024; Hilli & Pedersen, 2021; Pawils et al., 2023). Importantly, Pereira et al. (2023) noted that 100% of parents feel comfortable discussing health issues, including the prevention of harmful behaviors, with school nurses. Therefore, primary nurses should embrace their role as frontline health educators, have a strong ethical responsibility to prevent injury and disease, and have a responsibility to promote the well-being of younger children, adolescents, and their families. In line with the findings of this study, primary nurses are strongly encouraged to adopt an interdisciplinary approach to engaging with school principals, teachers, local policymakers, and parent representatives to develop comprehensive anti-smoking programs that leverage online technologies and culturally tailored strategies. Every change brings hope, but if we do nothing, nothing will change.

Furthermore, the findings revealed adolescent girls as significantly more willing than boys to advise family smokers. This may be influenced by traditional gender norms and socialization patterns that give greater encouragement to girls than boys to be expressive, empathetic, and communicative within the family context. The results of prior research suggest girls tend to possess stronger interpersonal skills and show a greater willingness than boys to engage in health-related discussions with parents and elders (Dieleman et al., 2021). In contrast, boys may feel constrained by societal expectations of masculinity that discourage emotional expression or confrontation, especially toward authority figures such as fathers. These gendered communication styles may partly explain why girls in this study were more proactive in applying the AFS strategies at home.

### Strengths and Limitations

A key strength of this study is the large sample size used and the high response rate, which allowed for adolescents’ experiences and challenges in advising family smokers after learning innovative strategies in a nurse-led AFS program to be explored fully. The scenario-based roleplay with the five steps of PDCAP may provide an effective care model for AFS in SHS prevention programs. However, this study was affected by several limitations. First, participants were drawn from a single rural county, which may limit the generalizability of the findings to other populations. Second, the use of retrospective self-reports and semi-structured questionnaires may introduce recall bias that skews the assessment of personal experiences and behaviors. Third, social desirability bias, especially regarding sensitive topics such as family smoking and adolescent attempts to advise smokers, may have influenced participants to provide responses they perceived as socially acceptable rather than fully accurate, which may have influenced the accuracy of reported AFS strategy use rates and perceived barriers. Future studies should consider incorporating anonymous digital tools or indirect questioning methods to reduce this bias and improve data reliability. Fourth, owing to participant anonymization, no additional analysis could be performed to compare successful and unsuccessful persuaders. Conducting such deeper analyses in future studies may yield important insights into refining future interventions.

### Conclusions

In this study, the high prevalence of SHS exposure among rural adolescents and the potential of a nurse-led innovative strategy designed to encourage and empower adolescent students to advise family members who smoke to help facilitate a smoke-free home environment were highlighted. While 46.9% of the participants attempted to advise family smokers, only 8.1% achieved a positive outcome, revealing both the promise and challenge of adolescent-led interventions. Given the cultural norms and authority dynamics prevalent among rural families, future research should explore long-term follow-up on the advisory behaviors of adolescents and examine the perspectives of targeted parents and guardians.
